# Reply to Wang: Policy failure can be assessed from observed outcomes

**DOI:** 10.1073/pnas.2617762123

**Published:** 2026-06-29

**Authors:** Yongyuan He, Yi Bu

**Affiliations:** ^a^ Department of Information Management, Peking University, Beijing 100871, People’s Republic of China; ^b^ Center for Informationalization and Information Management Research, Peking University, Beijing 100871, People’s Republic of China

We thank Wang for engaging with our work (1). The issues raised about policy timing, publication lag, and disclosure measurement are important, though most have been acknowledged in our article (2). However, they do not invalidate our central conclusion. Wang reads our title-level claim as an estimate of the causal effect of policy adoption for each journal; that is not the target of our study (1). We ask whether the current journal AI policy regime has produced restraint in AI-associated writing signals or public transparency. The evidence shows it has not.

Policy snapshots are an objective limitation, not an overlooked flaw. As we wrote, “the policy data were collected in two batches in January and October 2025,” and “the current release dates of the policies for each journal are lacking.” We also noted that “the academic publishing process involves several months or even longer,” and that “our results do not necessarily imply that the policies are ineffective, but their effects have not yet been fully reflected in the data” (2). Thus, we did not present a causal design based upon policy adoption dates. This limitation does not change the pattern: journals with AI policies did not show lower growth, and public disclosure remained rare.

We disagree that our conclusion of policy failure is unsupported. The claim is based on the aggregate evidence that journals with AI policies did not show slower growth than journals without such policies. It does not imply that every policy had no causal effect or that every AI-assisted manuscript violated journal rules. It points to a gap between policy adoption and outcomes.

Most journal AI policies are disclosure oriented rather than prohibitory in both rounds of policy collection. If journals permit AI-assisted writing but require disclosure, the central outcome is whether such use becomes visible. Public disclosure remained rare relative to estimated AI-assisted writing.

While we agree that full-text disclosure misses some disclosures made through submission systems or cover letters, our measure is public disclosure visible in published articles, not a complete audit of private channels. Disclosures invisible to readers do not provide public transparency.

Finally, our MLE-based measure should not be conflated with individual-level AI detection. It estimates AI-associated writing prevalence across large collections of papers, following Liang et al. (3, 4), a later study in *Science* (5), and many related work (6–9). As acknowledged, it cannot distinguish “linguistic polishing” from “substantive text generation” (2). This limitation affects interpretation, not the aggregate pattern. One should not use it to accuse authors of misconduct.

In sum, Wang identifies limitations we already recognized; and more importantly, these do not overturn the central gap: AI-associated writing has surged, journals with AI policies show no restraint, and public disclosure remains rare. This supports our conclusion that current journal AI policies have failed to deliver public transparency or restraint at scale.

## Data Availability

There are no data underlying this work.
